# Prevalence of Osteosynthesis Hardware Removal Due to Surgical Site Infections Following Sagittal Split Osteotomy: A Systematic Review and Meta-Analysis

**DOI:** 10.3390/jcm14103558

**Published:** 2025-05-19

**Authors:** Maria Kantzanou, Evangelos Kostares, Vasiliki Koumaki, Georgia Kostare, Michael Kostares, Athanasios Tsakris

**Affiliations:** 1 Department of Microbiology, Medical School, National and Kapodistrian University of Athens, 115 27 Athens, Greece; maria.kantzanou@gmail.com (M.K.); kostevang@med.uoa.gr (E.K.); vkoumaki@gmail.com (V.K.); gioulikostare@gmail.com (G.K.); 2 Department of Anatomy, Medical School, National and Kapodistrian University of Athens, 115 27 Athens, Greece; michkostares@gmail.com

**Keywords:** osteosynthesis hardware removal, surgical site infection, SSI, sagittal split ramus osteotomy, SSRO, prevalence, meta-analysis

## Abstract

**Background/Objectives:** Sagittal split ramus osteotomy (SSRO) is a commonly performed procedure in orthognathic surgery. Despite its effectiveness, surgical site infections (SSI) represent a significant postoperative complication, often necessitating the removal of osteosynthesis materials. This study aims to quantify the prevalence of hardware removal due to SSI following SSRO highlighting its impact on clinical outcomes. **Methods:** A systematic review and meta-analysis were conducted according to the PRISMA statement. Databases including Medline/PMC Central, Scopus, and Web of Science were searched up until 27 December 2024. Observational studies reporting osteosynthesis material removal due to SSI after SSRO were included. Data were extracted and analyzed using a random-effects model, calculating pooled prevalence and 95% confidence intervals (CI). Meta-regression was performed to explore potential predictors. **Results:** Twenty-nine studies published between 1992 and 2024 were included, encompassing 4489 patients. The pooled prevalence of osteosynthesis material removal due to SSI was 1.9% (95% CI: 0.7–3.4%), with substantial heterogeneity (I^2^ = 87%). Meta-regression demonstrated that the mean age of patients was significantly associated with the prevalence of osteosynthesis hardware removal due to SSI. On the other hand, no significant association was demonstrated between the year of publication, the proportion of males, or the mean age with the prevalence of removal. **Conclusions:** SSI following SSRO clearly impacts patient outcomes and healthcare resources, while removal of osteosynthesis materials is often required. The substantial heterogeneity among studies included in the present systematic review may point to variability in patient characteristics, surgical techniques, and healthcare practices. The present findings underscore the importance of standardized prevention protocols and targeted management strategies. Future research should focus on understanding microbial profiles, patient-specific risk factors, and innovative surgical approaches to minimize SSI risks and improve patient outcomes.

## 1. Introduction

Sagittal split ramus osteotomy (SSRO) is one of the most commonly performed osteotomies in orthognathic mandibular surgery, used to correct both anatomical and functional relationships in dentofacial deformities. This procedure involves splitting the mandibular ramus bilaterally to create separate proximal and distal bone segments, which are then repositioned to achieve proper occlusion and facial harmony. The bone fragments are typically stabilized using rigid internal fixation, most commonly titanium screws and plates. These osteosynthesis materials provide mechanical stability, promote optimal healing, and eliminate the need for prolonged intermaxillary fixation. However, the introduction of foreign materials into surgical sites carries inherent risks of complications, particularly surgical site infections (SSI), which can necessitate their removal.

Historically, Trauner and Obwegeser revolutionized mandibular surgery in 1957 with the introduction of the SSRO. In 1976, Spiessel proposed rigid internal fixation, a concept derived from orthopedic trauma surgery, to improve outcomes and eliminate the need for prolonged intermaxillary fixation lasting 5–6 weeks [1]. Among the indications for SSRO are mandibular excess, deficiency, and asymmetry. As with any surgical procedure, SSRO is associated with complications, including bleeding from injury to the inferior alveolar or masseteric arteries, fractures or “bad splits” (2.3% per SSRO) [2], surgical site infections (SSI) (9.6% per patient) [2], avascular necrosis, condylar resorption, worsening temporomandibular symptoms, inferior alveolar nerve injury, osteosynthesis material removal (11.2% per patient) [2], and lingual nerve injury, with a meta-analysis of 11 studies reporting a prevalence of 0.1% (95% CI 0.0–0.6%) [3]. Regarding the presence of inferior third molars during SSRO, De Souza B.B., et al. reviewed 19 articles analyzing unfavorable fractures, infection, neurosensory disturbances, osteosynthesis material removal, and surgery duration. No significant differences were observed in fracture rates (RR 0.95, 95% CI 0.58–1.57), infection (RR 0.75, 95% CI 0.48–1.18), or neurosensory disturbances (RR 1.55, 95% CI 0.61–3.91), although surgery duration was longer when third molars were present [4].

SSI are the most common nosocomial infections in surgical patients, significantly contributing to postoperative morbidity and mortality. Despite advancements, SSIs still account for over 2 million hospital-acquired infections annually in the United States. The Centers for Disease Control and Prevention (CDC) classifies SSI into superficial, deep incisional, and organ/space infections based on anatomical depth and clinical presentation. Diagnostic criteria include purulent discharge, positive microbial cultures, or clinical signs of infection occurring within 30 days of surgery or up to 90 days for implant-related procedures. SSI often result from contamination with endogenous or exogenous microbes, with risk factors including patient-related factors such as advanced age, obesity, diabetes, and smoking, as well as procedure-related factors such as prolonged surgical duration, inadequate asepsis, and improper antibiotic prophylaxis [4,5,6,7].

This systematic review and meta-analysis aims to quantify the prevalence of osteosynthesis material removal due to SSI following SSRO. This complication represents a significant postoperative challenge, adversely affecting patient outcomes and placing additional strain on healthcare resources. Although individual reports on this issue exist, consolidated data are scarce, limiting a comprehensive understanding of its burden and associated risk factors.

## 2. Materials and Methods

### 2.1. Eligibility Criteria

The eligibility of studies was determined using the PECOS framework [8], as follows:

**(P) Population**

- Inclusion Criteria: Patients aged over 18 years with dentofacial deformities.
- Exclusion Criteria: Pediatric population [9], studies with fewer than 30 patients [10,11,12], studies without clear documentation of the number of patients undergoing SSRO [13], and studies with overlapping populations.

**(E) Exposure**

- Inclusion Criteria: Patients undergoing SSRO, either as a standalone procedure or in combination with other orthognathic surgeries.
- Exclusion Criteria: Patients undergoing alternative mandibular orthognathic procedures (e.g., IVRO) [14,15,16], patients undergoing reoperation, patients solely with specific comorbidity, studies where the surgical procedure was not well-defined [17], studies including multiple fixation methods within the same cohort.

**(C) Comparator**

Not applicable.

**(O) Outcome**

- Inclusion Criteria: Hardware removal due to SSI.
- Exclusion Criteria: Studies mentioning only infection cases without hardware removal [18,19], studies not involving osteosynthesis material, studies without a clear definition of SSI [20], and cases from registries or multi-institutional databases [21,22].

**(S) Study Design**

- Inclusion Criteria: Observational studies written in English.
- Exclusion Criteria: Case reports and case series with fewer than 30 patients, systematic reviews, meta-analyses, narrative reviews, and other review articles, interventional studies, including randomized controlled trials (RCTs) and non-RCTs, conference abstracts, letters to the editor, expert opinion, retracted articles, articles with no full text available, and articles written in languages other than English [23].

### 2.2. Information Source

In accordance with the Cochrane Handbook for Systematic Reviews of Interventions, a thorough literature search was conducted. The PRISMA guidelines were used to structure and report this systematic review. The databases searched included Medline/PMC Central (via PubMed), Scopus, and Web of Science, as well as the reference lists of all identified relevant articles. The last search was conducted on 27 December 2024. The PRISMA checklist is available in the Supplementary Materials as Supplementary Table S1.

### 2.3. Search Strategy

The following search algorithm was used across the databases Medline/PMC Central (via PubMed), Scopus, and Web of Science:

- Medline/PMC Central: (mandib* OR lower jaw) AND (orthognathic OR corrective jaw OR bilateral sagittal split osteotom* OR osteotom*) AND (infect* OR sequal* OR complicat*), Filters: None
- Scopus: ((mandib* OR (lower AND jaw)) AND (orthognathic OR (corrective AND jaw) OR (bilateral AND sagittal AND split AND osteotom*) OR osteotom*) AND (infect* OR sequal* OR complicat*)), Filters: Title-Abstract-Keywords
- Web of Science: ((mandib* OR (lower AND jaw)) AND (orthognathic OR (corrective AND jaw) OR (bilateral AND sagittal AND split AND osteotom*) OR osteotom*) AND (infect* OR sequal* OR complicat*)), Filters: Articles, English language

### 2.4. Selection Process

Two independent reviewers applied the aforementioned search algorithm to identify relevant articles. Articles were collected, managed, and organized using Zotero (6.0.37) software. Duplicate articles were removed. The reviewers screened the remaining articles based on titles and abstracts. Full texts of potentially eligible articles were retrieved and carefully examined. Finally, the reviewers compared their selected articles, and any disagreements were resolved through team consensus.

### 2.5. Data Collection Process

The two reviewers independently extracted relevant data into an Excel spreadsheet. As the study is a proportional meta-analysis, the primary outcome was the overall incidence. Data collected included: author names, year of publication, study design, continent, country of origin, study period, total number of patients undergoing SSRO, proportion of male patients, mean age, total number of osteosynthesis material removals due to surgical site infections, any other significant details mentioned in the articles.

### 2.6. Study Risk of Bias Assessment

Most studies included were cohort designs (primarily retrospective). Two independent researchers assessed the risk of bias using the Newcastle–Ottawa Scale (NOS) and its adaptation for cross-sectional studies. For cohort studies, the NOS assesses three domains: (1) Selection, evaluating factors such as the representativeness of the exposed cohort, selection of the non-exposed cohort, ascertainment of exposure, and demonstration that the outcome was not present at baseline; (2) Comparability, evaluating whether the study controlled for key confounding factors; and (3) Outcome, assessing the adequacy of outcome ascertainment, sufficiency of follow-up duration, and completeness of follow-up. For cross-sectional studies, the NOS adaptation evaluates: (1) Selection (representativeness of the sample, sample size justification, comparability between respondents and non-respondents, and ascertainment of exposure with validated tools); (2) Comparability (control for the most important confounding factor and any additional factors); and (3) Outcome (assessment method—blind assessment, record linkage, or self-report—and appropriateness of the statistical analysis used). Disagreements between the two researchers during the risk of bias assessment were resolved through team consensus. Detailed scoring for each included study is presented in the Supplementary Material (Supplementary Table S2).

### 2.7. Effect Measure

The effect measure was expressed as a prevalence.

### 2.8. Statistical Analysis

Statistical analysis was conducted using RStudio (version 4.3.1) and the metafor package. The pooled prevalence and 95% confidence intervals (CI) were calculated using the DerSimonian and Laird random-effects model with Freeman–Tukey double arcsine transformation to account for extreme proportions observed in many studies [24].

Heterogeneity was assessed using Cochran’s Q statistic and the I^2^ statistic, with the following thresholds:

- 0–40%: Not significant
- 30–60%: Moderate
- 50–90%: Significant
- 75–100%: Substantial heterogeneity [25]

A sensitivity analysis was performed, and a meta-regression analysis was conducted for continuous variables (provided that more than 10 studies included relevant data) [26]. Statistical significance was set at *p* < 0.05. Publication bias was not assessed due to the nature of proportional studies, which are not comparable, and the inability to define positive results consistently [27].

## 3. Results

### 3.1. Study Selection

**Figure 1:**
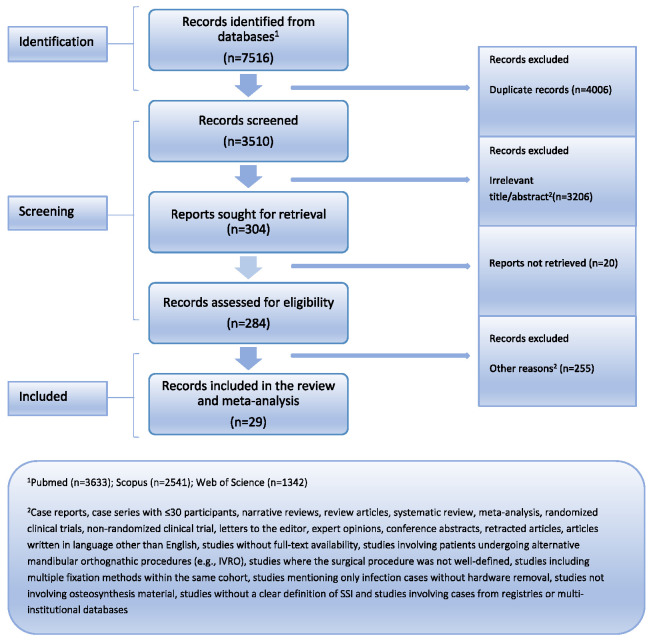
PRISMA flowchart.

A systematic search across Medline/PMC Central (via PubMed), Scopus, and Web of Science identified 7516 records, of which 3510 were screened after removing duplicates. Following title and abstract screening, 3206 records were excluded based on predefined criteria. Of 304 full-text reports sought, 20 were not retrieved, and 255 were excluded after assessment. Ultimately, 29 studies met the eligibility criteria and were included in the meta-analysis (Figure 1).

### 3.2. Study Characteristics

In total, 29 studies (n = 4489 patients) were included in this quantitative analysis, published between 1992 and 2024, with the studies conducted from 1992 to 2022. Most studies utilized a retrospective cohort design, while two were cross-sectional studies and one was a case series. Geographically, the majority of investigations were carried out in Europe (n = 20; Spain, Germany, Iceland, Finland, Austria, France, Norway, The Netherlands, Belgium, United Kingdom, Switzerland, Italy), followed by Asia (n = 6; Taiwan, Malaysia, Japan, Republic of Korea), North America (n = 2; United States, Canada), and South America (n = 1; Brazil). Collective analysis revealed that males constituted an average of 37.1% of the participants, with a mean age ranging from 22.0 to 35.1 years and a median mean age of 25.2 years. The unweighted positivity rate of osteosynthesis material removal due to infection ranged from 0% to 20%. Across the studies, infections were generally infrequent and predominantly localized. Not all reported infections necessitated the removal of osteosynthesis materials. For example, the study by Parente EV et al. [28], found that two patients experienced localized infections, both of which were successfully treated with oral systemic antibiotics, adherence to good oral hygiene, and application of chlorhexidine gel to the wound twice daily for 7 days, without requiring material removal. Another study reported four cases of SSI among 524 consecutive mandibular SROs (4/524, 0.8%); none of these cases required the removal of the fixation hardware [29]. In contrast, in the studies by Kuhlefelt M., et al. [30], Theodossy T. et al. [31], and Mohamad NH., et al. [31], all infected patients underwent osteosynthesis material removal. Follow-up durations varied considerably among the studies, ranging from as short as 1.5 months to as long as 24 months, though the majority of studies had a minimum follow-up of 12 months [32,33,34,35,36,37,38,39,40,41], allowing for diverse assessments of both short- and long-term outcomes. Quality assessment classified all the studies as moderate (Supplementary Table S2). The descriptive characteristics of these studies are summarized in Table 1.

### 3.3. Results of Syntheses

**Figure 2:**
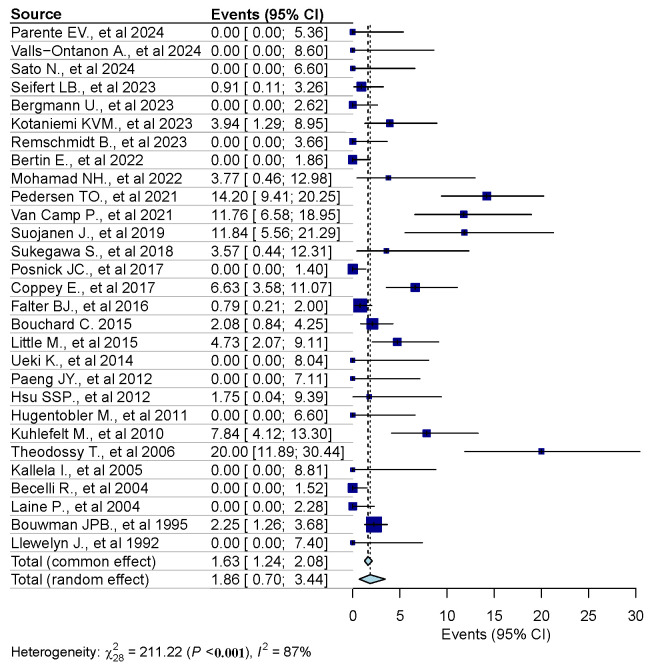
Forest plot [28,29,30,31,32,33,34,35,36,37,38,39,40,41,42,43,44,45,46,47,48,49,50,51,52,53,54,55,56].

**Figure 3:**
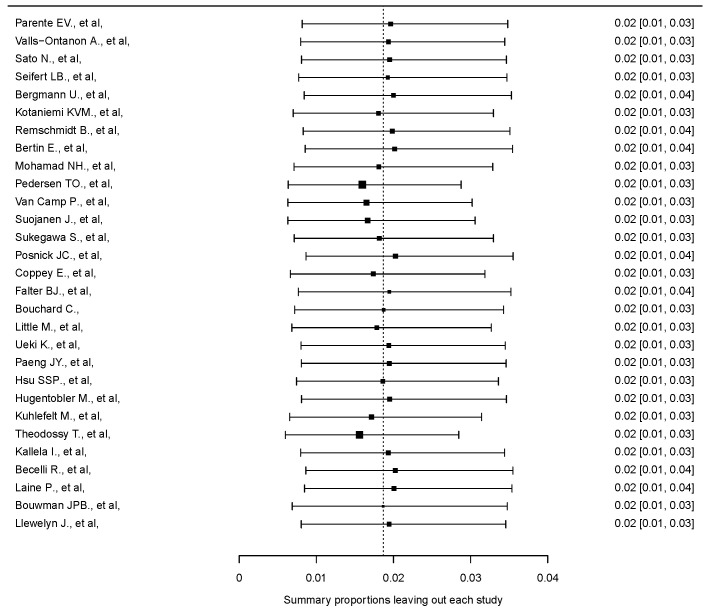
Leave one out analysis [28,29,30,31,32,33,34,35,36,37,38,39,40,41,42,43,44,45,46,47,48,49,50,51,52,53,54,55,56].

**Figure 4:**
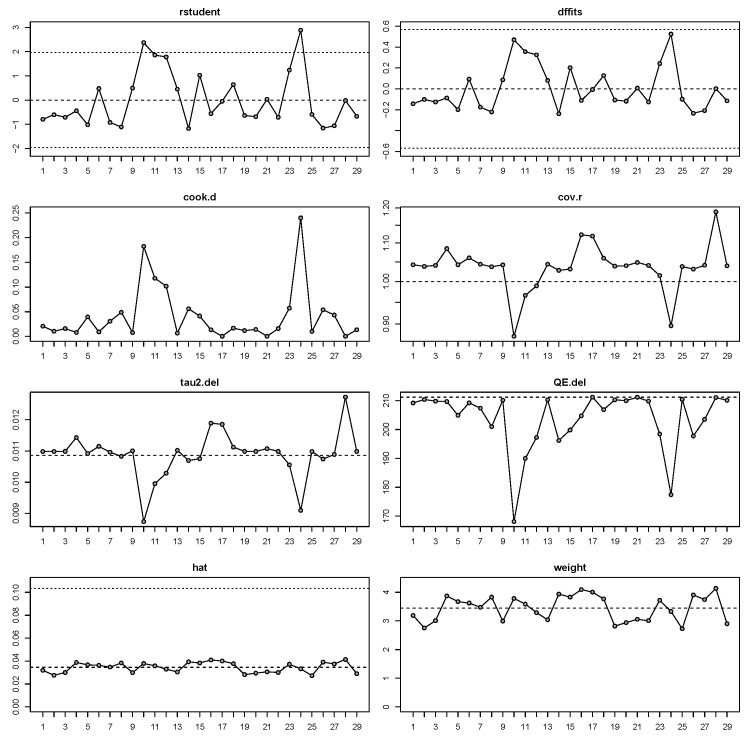
Influential diagnostics.

The random-effects model analysis estimated the prevalence of osteosynthesis material removal due to SSI following SSRO at 1.9% (95% CI: 0.7–3.4%), with substantial heterogeneity observed among the included studies (I^2^ = 87%, 95% CI: 81–93%) (Figure 2). Diagnostic analyses and a forest plot demonstrating the results of the leave-one-out sensitivity analysis are provided as Figure 3 and Figure 4. These analyses did not identify any study as influential.

### 3.4. Meta-Regression Analysis

Meta-regression analysis showed no significant associations between the prevalence of hardware removal due to SSI and mean patient age, year of publication, or proportion of males (Supplementary Materials, Supplementary Table S3).

## 4. Discussion

This review is meant to provide a fundamental baseline for this estimation of the overall prevalence of osteosynthesis material removal due to SSI following SSRO. The estimated overall prevalence is calculated as 1.9% (95% CI: 0.7–3.4%), with substantial heterogeneity observed among the included studies. Through the meta-regression analysis, we demonstrated that the mean age of patients was significantly associated with the prevalence of osteosynthesis hardware removal due to SSI. In contrast, no significant associations were observed for the year of publication or the proportion of males. Zirk M., et al. (2023) [57] reported a prevalence of 2.4% for osteosynthesis-associated infections (OAI) across a diverse set of maxillofacial surgical procedures, emphasizing the role of bacterial biofilms in mediating infection and the subsequent necessity for implant removal. They highlighted that larger volumes of osteosynthetic material, particularly reconstruction plates, were associated with a higher risk of infection compared to smaller implants used in procedures like orthognathic surgery. Moreover, their study demonstrated anatomical variability, with mandibular sites exhibiting a higher susceptibility to infection, reflecting the unique biomechanical and microbiological challenges in this region. Moreover, findings that patient-specific factors, such as multimorbidity and comorbid conditions like diabetes mellitus, further amplify infection risks [57]. However, we could not include these variables in the meta-regression analysis due to fewer than 10 studies addressing them in the identified articles. The interplay between host immune response, implant material properties, and the microbial environment underscores the multifactorial etiology of SSIs. Furthermore, Zirk M., et al. underscore the significance of antibiotic regimens targeting specific pathogens, noting that *Streptococcus* spp., *Prevotella* spp., *Staphylococcus* spp., and *Veillonella* spp. are commonly associated with infections in smaller implant volumes, while E. faecalis, *P. mirabilis*, and *P. aeruginosa* are more prevalent in infections involving larger implant volumes [57]. Vishal R., et al. (2020) [58] explored infections following open reduction and internal fixation (ORIF) for maxillofacial fractures, predominantly identifying infections in the mandibular region. The study highlighted the dominance of *Staphylococcus aureus* (50%) among the isolated bacteria, followed by *Pseudomonas aeruginosa*, *Escherichia coli*, and *Streptococcus salivarius*, reflecting the diverse and region-specific microbial milieu in craniomaxillofacial infections. These findings emphasize the importance of routine microbial analyses and antibiotic susceptibility testing for effective management. While our meta-analysis did not include data on microbial profiles, the bacteria reported by Vishal et al. underscore the potential involvement of similar pathogens in infections following orthognathic surgeries [58,59].

Regarding the use of biodegradable osteosynthesis materials, Gareb B., et al. (2021) [60] investigated the outcomes of biodegradable versus titanium osteosynthesis systems in orthognathic surgery. They highlighted that titanium systems, while effective, are often removed due to complications such as plate palpability, pain, and potential inflammatory responses, with removal rates reaching up to 33% for titanium systems and 17% for biodegradable systems. Biodegradable systems emerged as an alternative with comparable symptomatic device removal rates (RR 1.29; 95% CI: 0.68–2.44). However, they noted an increased operative time in the biodegradable group (SMD 0.50; 95% CI: 0.09–0.91) and potential for material-related issues such as foreign body reactions. Implant characteristics and patient-specific factors play crucial roles in surgical outcomes, further emphasizing the need for personalized approaches to osteosynthesis [60].

Gómez-Barrachina R., et al. (2020) [61] performed a systematic review and meta-analysis focusing on titanium plate removal in orthognathic surgeries. They reported an overall prevalence of plate removal at 13.4% (95% CI: 9.6–18.3%) and identified infection as the primary cause, accounting for 6.6% of cases. They also noted higher risks of plate removal associated with mandibular placements, smoking, and female sex, indicating significant demographic and anatomic variability in infection susceptibility. Despite the absence of microbial data in our meta-analysis, these findings reiterate the importance of targeted preventive strategies and individualized patient management to mitigate risks associated with surgical interventions in orthognathic procedures [61].

Our systematic review and meta-analysis is not without limitations. First, it should be noted that we utilized observational studies, most of which were retrospective in nature. Heterogeneity remains considerable. These studies were conducted at different times and in diverse settings, each following its own institutional directives and baselines, which influenced factors ranging from the definition of SSI to the management of these infections. For instance, in many studies, osteosynthesis material was removed as part of the treatment for SSI, while in others, cases were managed conservatively with antibiotics or other measures without removing the osteosynthesis material. Different mean of internal fixation was used. Differences in antibiotic prophylaxis protocols and other factors may have further influenced outcomes. Additionally, we included a few studies where the evidence for the lack of osteosynthesis material removal due to SSI was indirect or not clearly stated. However, studies that did not clearly describe or provide acceptable evidence for the management of osteosynthesis materials were excluded. Only studies published in English were considered. Moreover, the data transformation methods employed in our analysis have recently been criticized [24], which might have implications for the interpretation of results. All studies were assessed as being of moderate quality. Moreover, our meta-analysis has not been registered in PROSPERO, which may be a source of bias.

## 5. Conclusions

The removal of osteosynthesis materials due to SSI following SSRO remains a clinically significant complication, with a pooled prevalence of 1.9% observed in this systematic review and meta-analysis. These findings highlight the impact of SSI on patient outcomes and healthcare resources. The substantial heterogeneity among studies underscores the need for standardized protocols and further research to identify modifiable risk factors, microbial profiles, and patient-specific characteristics to improve management strategies.

## Figures and Tables

**Table 1:** Study characteristics.

Authors	Year of Publication	Study Design	Continent of Origin	Country	Study Period	Total Patients	Proportion of Males	Mean Age	Osteosynthesis Materials Removal Due to SSI	Removal Rate Due to Infection (%)	Quality Assessment
Parente EV., et al. [28]	2024	Cross-sectional	South America	Brazil	2020–2022	67	28.4	34	0	0	Moderate
Valls-Ontanon A., et al. [33]	2024	Cohort	Europe	Spain	2018–2019	41	46.3	30.8	0	0	Moderate
Sato N., et al. [34]	2024	Cohort	Asia	Taiwan	2018–2021	54	33.3	22	0	0	Moderate
Seifert LB., et al. [35]	2023	Cohort	Europe	Germany	2009–2019	219	42.4	25.2	2	0.9	Moderate
Bergmann U., et al. [42]	2023	Cohort	Europe	Iceland	2010–2022	139	NA ^1^	NA ^1^	0	0	Moderate
Kotaniemi KVM., et al. [43]	2023	Cohort	Europe	Finland	2006–2020	127	40.2	30	5	3.9	Moderate
Remschmidt B., et al. [44]	2023	Cohort	Europe	Austria	2017	99	31.3	30.1	0	0	Moderate
Bertin E., et al. [45]	2022	Cohort	Europe	France	2012–2022	197	NA ^1^	NA ^1^	0	0	Moderate
Mohamad NH., et al. [32]	2022	Cross-sectional	Asia	Malaysia	2011–2017	53	NA ^1^	NA ^1^	2	3.8	Moderate
Pedersen TO., et al. [46]	2021	Cohort	Europe	Norway	2013–2019	176	37.5	NA ^1^	25	14.2	Moderate
Van Camp P., et al. [36]	2021	Cohort	Europe	The Netherlands	2017–2018	119	NA ^1^	NA ^1^	14	11.8	Moderate
Suojanen J., et al. [37]	2019	Cohort	Europe	Finland	NA ^1^	76	NA ^1^	NA ^1^	9	11.8	Moderate
Sukegawa S., et al. [47]	2018	Cohort	Asia	Japan	2003–2017	56	NA ^1^	NA ^1^	2	3.6	Moderate
Posnick JC., et al. [29]	2017	Cohort	North America	USA	2004–2013	262	48.9	25	0	0	Moderate
Coppey E., et al. [48]	2017	Cohort	Europe	Belgium	2012–2015	196	41.3	26.1	13	6.6	Moderate
Falter BJ., et al. [38]	2016	Cohort	Europe	Belgium	2010–2012	509	36.3	26.3	4	0.8	Moderate
Bouchard C., et al. [49]	2015	Case-series	North America	Canada	2008–2013	336	26.5	27.2	7	2.1	Moderate
Little M., et al. [50]	2015	Cohort	Europe	United Kingdom	2004–2012	169	NA ^1^	NA ^1^	8	4.7	Moderate
Ueki K., et al. [39]	2014	Cohort	Asia	Japan	NA ^1^	44	36.4	29.1	0	0	Moderate
Paeng JY., et al. [40]	2012	Cohort	Asia	Republic of Korea	NA ^1^	50	48	NA ^1^	0	0	Moderate
Hsu SSP., et al. [51]	2012	Cohort	Asia	Taiwan	200–2004	57	45.6	NA ^1^	1	1.8	Moderate
Hugentobler M., et al. [52]	2011	Cohort	Europe	Switzerland	NA ^1^	54	38.9	25.9	0	0	Moderate
Kuhlefelt M., et al. [30]	2010	Cohort	Europe	Finland	1997–2003	153	41	35.1	12	7.8	Moderate
Theodossy T., et al. [31]	2006	Cohort	Europe	United Kingdom	2001–2003	80	26.3	25	16	20	Moderate
Kallela I., et al. [53]	2005	Cohort	Europe	Finland	NA ^1^	40	27.5	29	0	0	Moderate
Becelli R., et al. [41]	2004	Cohort	Europe	Italy	1996–2001	241	32.8	24	0	0	Moderate
Laine P., et al. [54]	2004	Cohort	Europe	Finland	NA ^1^	160	NA ^1^	NA ^1^	0	0	Moderate
Bouwman JPB., et al. [55]	1995	Cohort	Europe	The Netherlands	NA ^1^	667	NA ^1^	NA ^1^	15	2.2	Moderate
Llewelyn J., et al. [56]	1992	Cohort	Europe	United Kingdom	NA ^1^	48	33	23.6	0	0	Moderate

^1^ NA: not applicable.

## Data Availability

Literature and Rstudio data are available from the corresponding author upon reasonable request.

## Supplementary Materials

The following supporting information can be downloaded at: https://www.mdpi.com/article/10.3390/jcm14103558/s1, Table S1: PRISMA checklist; Table S2: Quality assessment; Table S3: Meta-regression analysis.

## Abbreviations

The following abbreviations are used in this manuscript:

SSRO	Sagittal split ramus osteotomy
SSI	Surgical site infection
IVRO	Intraoral vertical ramus osteotomy
RR	Risk ratio
CDC	Centers for Disease Control and Prevention
PRISMA	Preferred Reporting Items for Systematic reviews and Meta-Analyses
CI	Confidence intervals
RCT	Randomized controlled trials
ORIF	Open reduction and internal fixation
OIA	Osteosynthesis-associated infections
